# Dialysed Iron in Arsenical Poisoning

**Published:** 1878-03

**Authors:** R. V. Mattison


Dialysed Iron in Arsenical Poisoning.—The administration of dialysed iron as an antidote for arsenical poisoning should be followed immediately by a teaspoonful or more of common salt, thus insuring the formation of the ferric hydrate and the consequent neutralization of the poison. This should at once be followed by an emetic, as the action of the ferric hydrate on the arsenic is not to coagulate it, but to form a perfectly definite chemical salt known as the arsenite of iron (ferric arsenite), which, though practically insoluble, is far from being harmless.—*R. V. Mattison, Ph. G., in Phila. Med. Times.*

